# Risk predictive model based on three immune-related gene pairs to assess prognosis and therapeutic sensitivity for hepatocellular carcinoma

**DOI:** 10.1186/s12957-022-02681-4

**Published:** 2022-08-05

**Authors:** Baifeng Qian, Haozhong Lin, Tian Lan, Muqi Li, Xiwen Wu, Shuirong Lin, Zimin Song, Shunli Shen, Baogang Peng

**Affiliations:** Center of Hepato-Pancreato-Biliary Surgery, The First Affiliated Hospital of Sun Yat-sen University, Guangzhou, Guangdong China

**Keywords:** Hepatocellular carcinoma, Prognosis, Risk predictive model, Immunotherapy, Anti-angiogenesis targeted therapy

## Abstract

**Background:**

Hepatocellular carcinoma (HCC) as a common tumor has a poor prognosis. Recently, a combination of atezolizumab and bevacizumab has been recommended as the preferred regimen for advanced HCC. However, the overall response rate of this therapy is low. There is an urgent need to identify sensitive individuals for this precise therapy among HCC patients.

**Methods:**

The Wilcox test was used to screen the differentially expressed immune-related genes by combining the TCGA cohort and the Immunology Database. Univariate and multivariate Cox regression analysis were used to screen the immune gene pairs concerning prognosis. A predictive model was constructed using LASSO Cox regression analysis, and correlation analysis was conducted between the signature and clinical characteristics. ICGC cohort and GSE14520 were applied for external validations of the predictive risk model. The relationship between immune cell infiltration, TMB, MSI, therapeutic sensitivity of immune checkpoint inhibitors, targeted drugs, and the risk model were assessed by bioinformatics analysis in HCC patients.

**Results:**

A risk predictive model consisting of 3 immune-related gene pairs was constructed and the risk score was proved as an independent prognostic factor for HCC patients combining the TCGA cohort. This predictive model exhibited a positive correlation with tumor size (*p* < 0.01) and tumor stage (TNM) (*p* < 0.001) in the chi-square test. The predictive power was verified by external validations (ICGC and GSE14520). The risk score clearly correlated with immune cell infiltration, MSI, immune checkpoints, and markers of angiogenesis.

**Conclusions:**

Our research established a risk predictive model based on 3 immune-related gene pairs and explored its relationship with immune characteristics, which might help to assess the prognosis and treatment sensitivity to immune and targeted therapy of HCC patients.

**Supplementary Information:**

The online version contains supplementary material available at 10.1186/s12957-022-02681-4.

## Introduction

Hepatocellular carcinoma (HCC), as the third leading cause of cancer-related mortality worldwide [1], has a poor prognosis with an average 5-year survival rate of 19.6% according to the National Cancer Institute’s Surveillance, Epidemiology, and End Results (SEER) database [2]. Although liver resection and liver transplantation keep the median overall survival at more than 6 years for early-stage patients [3], high recurrence rates could reach 40–70% within 5 years [4, 5]. Systemic therapy with the drugs of atezolizumab (anti-PD-L1) and bevacizumab (anti-VEGFA), as a standard first-line therapy for advanced HCC according to the clinical practice guideline [6], showed a median overall survival (OS) time that was double that of sorafenib and significantly improved the 12-month OS in the clinical trial IMbrave150 [7]. However, the overall response rates (ORR) of the combination therapy and monotherapy of atezolizumab were 20% and 17%, respectively [8]. The majority of HCC patients are insensitive to these therapies.

Several factors, such as tumor mutational burden (TMB), microsatellite instability (MSI), play an important role in tumor prognosis. MSI and TMB could help to screen patients who may benefit from immune checkpoint inhibitor (ICI) therapy [9, 10]. However, their status in HCC has not been well defined [11, 12]. It is urgent to find a better biomarker to identify patients who might benefit from targeted and ICI therapies.

Our study constructed a risk predictive model composed of 3 immune-related gene (IRG) pairs for predicting prognosis, characterizing immune cell infiltration, evaluating the relationship with TMB and MSI, and assessing the therapeutic sensitivity of ICIs and small molecular targeted drugs in HCC patients.

## Materials and methods

### Training and validating data collection

The training group that included 365 patients with RNA sequencing data, clinical information, and simple nucleotide variations were obtained from The Cancer Genome Atlas (TCGA-LIHC, https://www.cancer.gov/tcga). RNA sequencing data and clinical information for external validations that included 231 patients from the International Cancer Genome Consortium (ICGC, LIRI-JP, https://dcc.icgc.org) and 242 patients from GEO database (GSE14520, https://www.ncbi.nlm.nih.gov/geo).

### Identifying the differential expression IRGs

A comprehensive list of IRGs was obtained from the Immunology Database and Analysis Portal database (https://immport.niaid.nih.gov/home) [13]. Then, IRGs in the training group were screened out by making an intersection with the Immunology Database. The R package “*edgeR*” was used to screen differentially expressed IRGs between tumor and normal tissues with the filter criteria (false discovery rate (FDR) < 0.05 and absolute log2 foldchange (|logFC|) > 2). Results are shown by heatmap and volcano plot using the “pheatmap” package and “limma” package.

### Screening IRG pairs and constructing a risk predictive model

By comparing the expression levels between the two IRGs in gene pairs, we define all gene pairs as either 1 or 0. The filter criterion is that 1 or 0 accounts for no more than 80% of all samples; otherwise, these gene pairs will be eliminated.

We screened the immune gene pairs using univariate Cox regression analysis related to prognosis (*p* < 0.001). These IRG pairs were included in the construction of a risk model using the least absolute shrinkage and selection operator (LASSO) Cox regression. The multivariate Cox regression analysis (*p* < 0.05) was performed to shrink the size of IRG pairs with a non-zero regression coefficient (β). Then the IRG Pairs were used to construct a risk score model to assess the sensitivity of ICIs and targeted therapy. The patients were divided into groups with high and low risk by optimal cutoff. Until the expressions of PD-L1 and VEGFA between high and low risk score groups showed a statistically significant difference, respectively (*p* < 0.01), the final IRG pairs were identified and a risk predictive model was established. The time-dependent ROC curves of the risk score were generated for estimating the model’s predictive power using the “survivalROC” package.

### Accessing the model’s predictive power

We conducted a Kaplan-Meier (KM) analysis to reflect the OS of both groups using the “survival” package in R. The ROC curves combined with clinical characteristics, which showed the risk model’s predicting power for prognosis, were demonstrated using the “survival ROC” package. In addition, we analyzed the correlation between the risk score and several clinical characteristics, including age, gender, tumor stage [tumor–node–metastasis (TNM)], pathological grade, cirrhosis, HBV infection, and recurrence status.

### External validations

ICGC cohort and GSE14520 were used for the external validations. The Independence validation of the risk score of the model was examined through univariate and multivariate Cox regression analyses using ICGC and GSE14520. Patients were classified into high and low-risk groups combined with the optimal cutoff value. KM survival analyses and time-dependent ROC curves were produced based on the ICGC and GSE14520. We also performed correlation analyses between the risk score and clinical information, including age, gender, tumor stage, pathological grade, cirrhosis, HBV infection, and recurrence status, to test the predictive power of this model.

### Identifying MSI and TMB characteristics

The MSI and TMB of every sample were obtained by using simple nuclear variations data in TCGA-LIHC. The optimal cutoffs of the MSI and TMB were investigated associated with survival outcomes. The cutoff values of the MSI and TMB were used to divide patients into high and low groups, respectively. Combined with the risk score, KM survival analyses were created to reflect the OS of the subgroup. The percentage of patients with different MSI or TMB in the high and low-risk groups was shown by bar plot. The correlation analyses between the risk score and MSI or TMB were explored.

### Assessment of immune infiltration, immunotherapy, and target therapeutic sensitivity

To evaluate the immune infiltration in different risk groups, the CIBERSORT algorithm was used to transform the expression profile into 22 immune cell infiltrations [14]. The Wilcoxon rank-sum test was performed to compare the difference between the two groups concerning the immune infiltrate abundance. To assess the sensitivity of ICI therapy and target therapy, different expressions of ICIs and markers associated with angiogenesis were compared between the two groups.

## Results

### Screening differentially expressed IRGs and IRG pairs

**Fig. 1:**
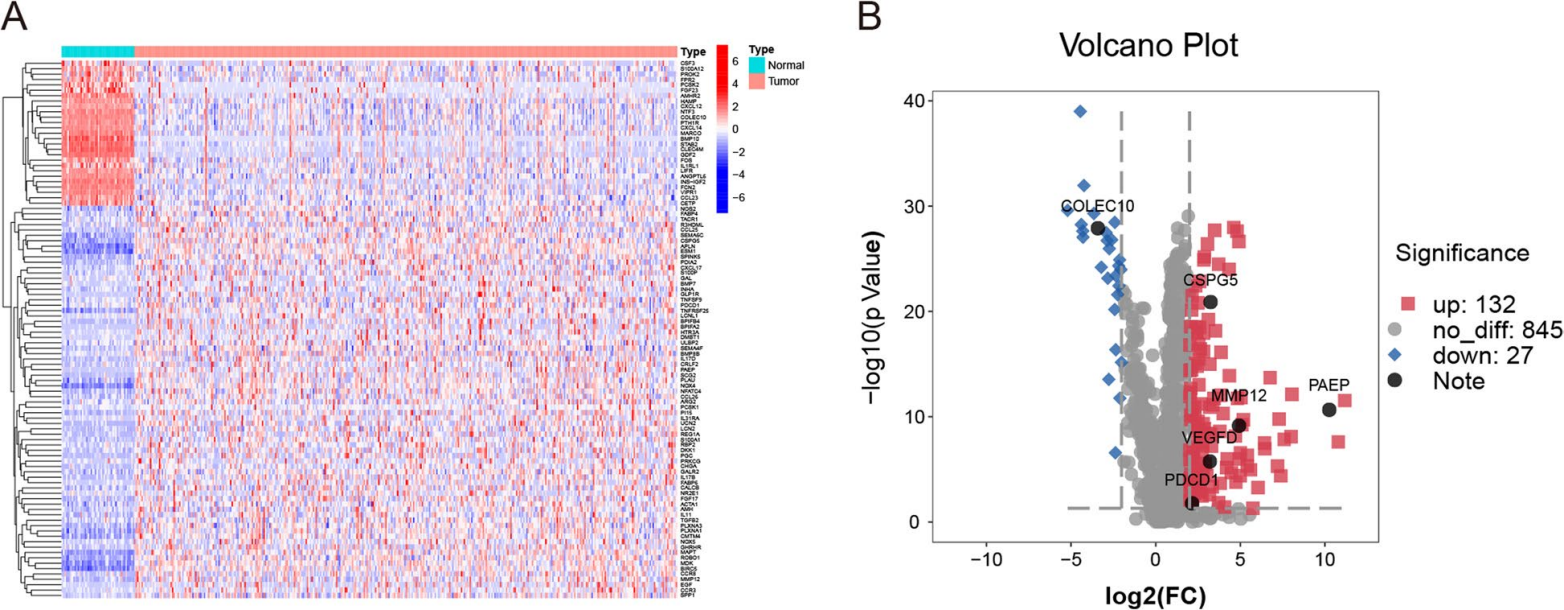
Differentially expressed immune genes of HCC tissues and normal tissues. **A** Heatmap of 100 top-ranked immune genes of tumor tissues and normal tissues. **B** Volcano plot of differentially expressed immune genes. Blue, downregulated; red, upregulated; Note: Collectin subfamily member 10, COLEC10; human matrix metallopeptidase 12, MMP12; progesterone associated endometrial protein, PAEP; chondroitin sulfate proteoglycan 5, CSPG5; programmed cell death 1, PDCD1; vascular endothelial growth factor D, VEGFD

One hundred fifty-nine differential expression IRGs were obtained. They included 27 downregulated and 132 upregulated genes (Fig. 1A, B). After pairing 159 IRGs, 5754 IRG pairs were included for univariate Cox regression analysis related to the OS. One hundred fifty-three prognostic IRG pairs were obtained, and in which 117 IRG pairs were prognostic risk factors (hazard ratio > 1) and others were prognostic protective factors (hazard ratio < 1). After using the LASSO Cox regression and multivariate Cox regression analysis were performed with 153 IRG pairs, 3 IRG pairs were identified, and both expressions of PD-L1 and VEGFA between high and low risk score groups showed significant difference.

### Risk predictive model construction for IRG pairs

**Fig. 2:**
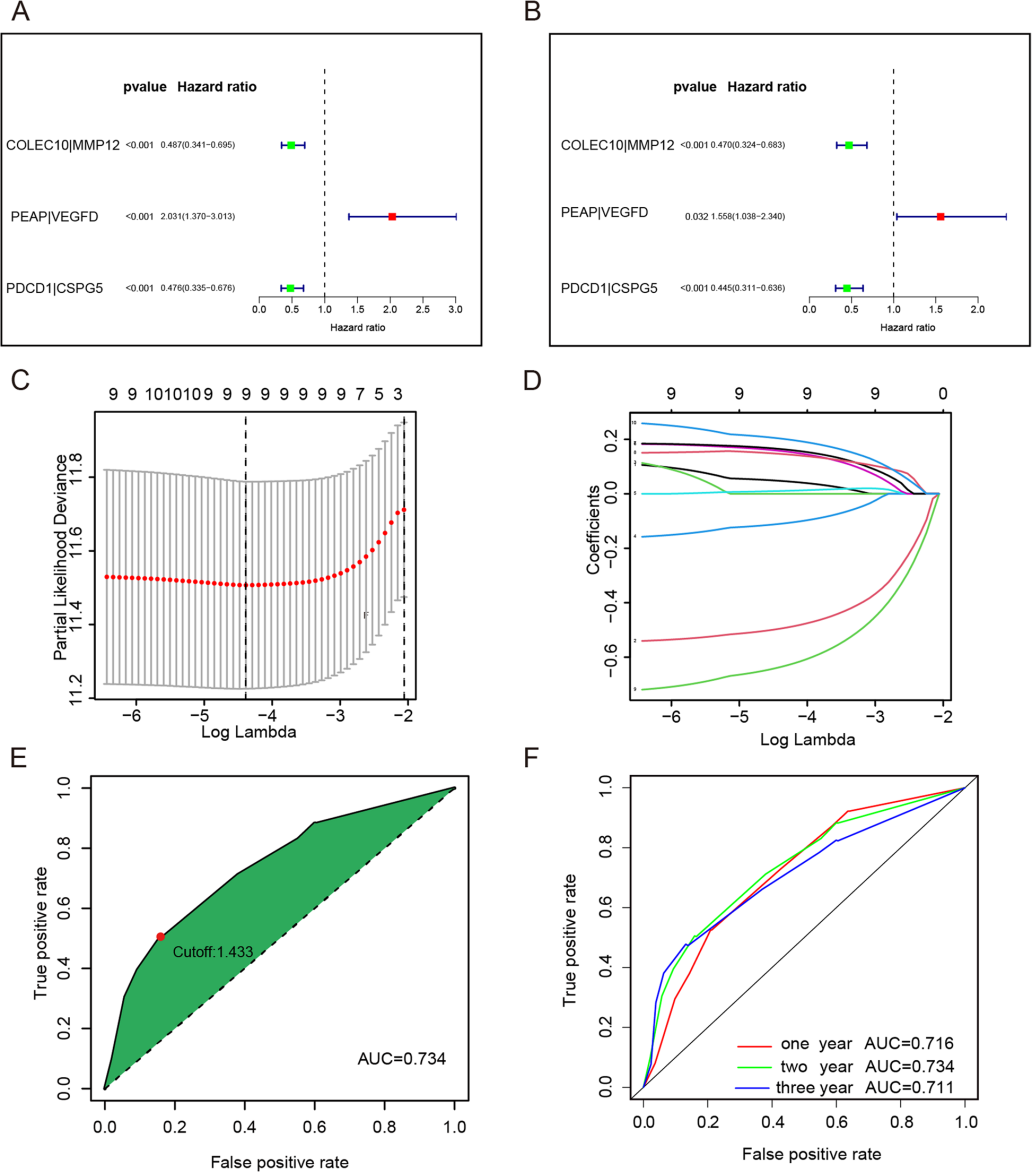
Construction of the risk predictive model using the differentially expressed IRG pairs. Univariate Cox regression analysis (**A**) and multivariate analysis (**B**) to determine the risk effects of IRG pairs in the TCGA dataset. **C**, **D** The establishment of the prognostic model based on LASSO penalized Cox analysis. **E** The optimal cutoff (1.433) of the risk model was used to classify patients into low- and high-risk groups. **F** Time-dependent ROC analysis for predicting the overall survival of patients in the TCGA cohort using the risk score

**Fig. 3:**
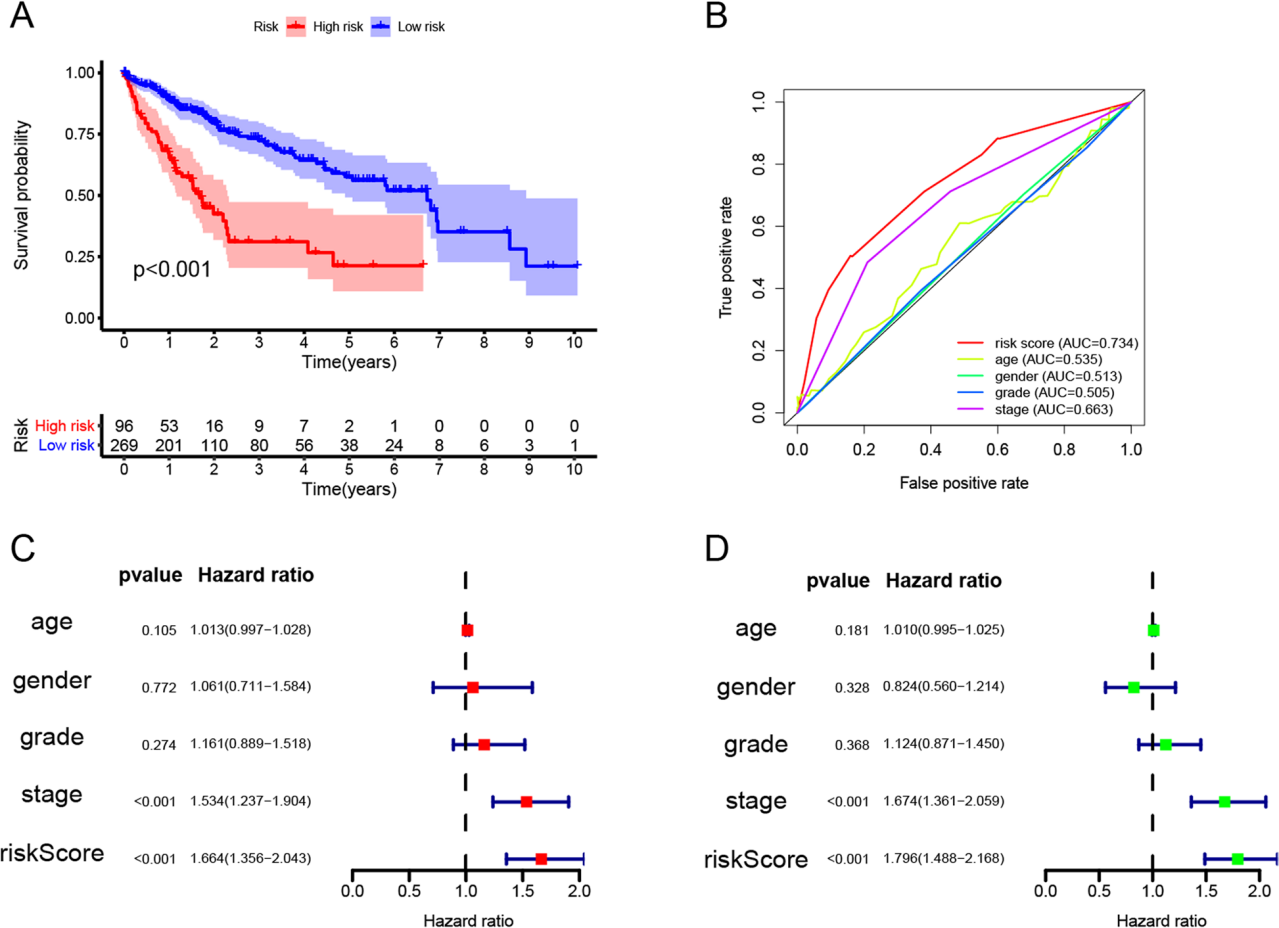
The risk model’s predictive power in the TCGA cohort. **A**, **B** Kaplan-Meier survival analysis and ROC analysis based on clinical characteristics for predicting the overall survival of patients using the risk score. Univariate Cox regression analysis (**C**) and multivariate analysis (**D**) to identify independent prognostic factors for overall survival based on clinical characteristics and risk score

The identified 3 IRG pairs were used to construct a LASSO Cox regression prognostic model (Fig. 2A–D). The patient’s risk score was calculated using the following formula: (COLEC10|MMP12 × − 0.755046442) + (PAEP|VEGFD × 0.443541094) + (PDCD1|CSPG5 × − 0.810587342). Three hundred sixty-five patients were classified into the high and low risk score groups with the optimal cutoff (1.433) of the risk score (Fig. 2E). The areas under the curve (AUC) of the risk score for predicting OS at 1, 2, and 3 years were 0.716, 0.734, and 0.711, respectively (Fig. 2F). The KM survival analysis showed the OS was clearly lower in the high-risk group (Fig. 3A). Based on the risk predictive model, the risk score is a better marker than age, gender, tumor stage, and pathological grade (Fig. 3B). Based on the univariate and multivariate Cox regression analyses, the risk score could be treated as an independent factor for predicting the prognosis, and its predictive power is better than that of tumor stage (Fig. 3C, D). In more detailed analyses with clinical information, tumors with larger size or in more advanced stages were found at higher risk scores (Table 1). These results showed that the risk score is effective to stratify the prognosis of patients.

**Table 1:** The chi-square test of the relation between risk score and clinical features in TCGA cohort

Clinical features	TCGA, n = 365		
	High risk	Low risk	*P*
Survival status			< 0.001
Survived	45	194	
Died	51	75	
Age			0.89
≥ 60 years	52	148	
< 60 years	44	121	
Sex			0.23
Male	60	186	
Female	36	83	
Histological grade			0.06
G1–2	53	177	
G3–4	42	88	
Gx	1	4	
Stage			< 0.001
I–II	55	199	
III–IV	35	52	
x	6	18	
T classification			< 0.01
T1–2	61	210	
T3–4	35	56	
Tx	0	3	
N classification			0.30
N0	66	182	
N1	2	2	
Nx	28	85	
M classification			0.12
M0	69	194	
M1	2	1	
Mx	25	74	
Cirrhosis status			0.74
No cirrhosis	16	61	
Cirrhosis	30	102	
Unknown	50	106	
Recurrence status			0.38
No recurrence	35	117	
Recurrence	43	114	
Unknown	18	38	
Hepatitis status			0.81
Hepatitis B	21	67	
Hepatitis C	11	32	
No hepatitis	64	170	

The cases with “Gx,” “Tx,” “Nx,” “Mx,” stage “x,” cirrhosis status “unknown,” and recurrence status “unknown” were excluded from the chi-square test

### External validations of the risk predictive model

**Fig. 4:**
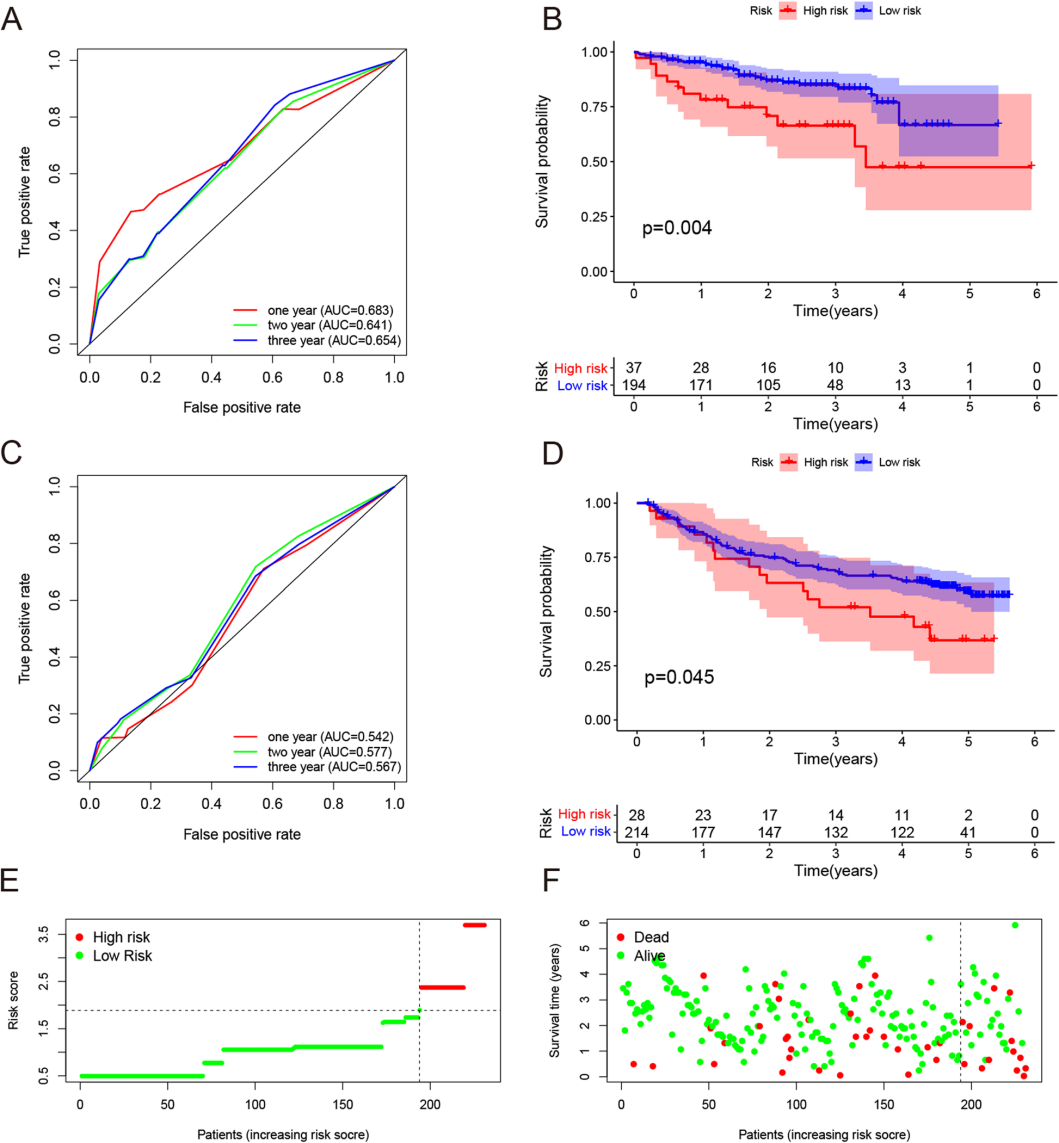
External validation of the risk predictive model in the ICGC and GSE14520. **A**, **B** The time-dependent ROC analysis and Kaplan-Meier survival analysis for predicting the overall survival of patients using the risk score in the ICGC cohort. **C**, **D** The time-dependent ROC analysis and Kaplan-Meier survival analysis for predicting the overall survival of patients using the risk score in the GSE14520. **E** Distribution of the risk score. **F** Survival status of patients

On external validations of ICGC and GSE14520, the risk predictive model showed good quality of applicability and stability to predict the prognosis of HCC. The AUC curves of the risk model for predicting OS in the ICGC cohort at 1, 2, and 3 years were 0.683, 0.641, and 0.654, respectively (Fig. 4A). While, in GSE14520 the AUC curves of the risk model for predicting the OS at 1, 2, and 3 years were 0.542, 0.577, and 0.567, respectively (Fig. 4C). Both the KM survival analyses presented a clearly lower OS in the high-risk group (Fig. 4B, D). The number of deaths increased with the increasing risk score (Fig. 4E), and the high-risk group had a poor prognosis (Fig. 4F). Moreover, tumors with a larger size or higher grade corresponded to a higher risk score in the ICGC cohort (Table 2). However, the cirrhosis, HBV infection, and recurrence status in GSE14520 cohort did not reach significant difference in correlation analysis with risk score (Table 2).

**Table 2:** The chi-square test of the relation between risk score and clinical features in ICGC and GSE14520 cohort

Clinical features	ICGC, *n* = 231			GSE14520, *n* = 242		
	High risk	Low risk	*P*	High risk	Low risk	*P*
Survival status			0.04			0.04
Survived	43	146		12	134	
Died	16	26		16	80	
Age			0.63			0.17
≥ 60 years	49	138		3	47	
< 60 years	10	34		25	167	
Sex			0.84			0.12
Male	44	126		27	184	
Female	15	46		1	30	
Stage			< 0.001			0.08
I–II	25	116		18	156	
III–IV	34	56		10	41	
x	0	0		0	17	
Histological grade			< 0.01			
G1–2	30	128				
G3–4	21	33				
Gx	8	11				
Cirrhosis status						0.37
No cirrhosis				1	18	
Cirrhosis				27	196	
Recurrence status						0.19
No recurrence				9	97	
Recurrence				19	117	
Hepatitis status						0.23
Hepatitis B				27	191	
No hepatitis				1	23	

The cases with “Gx” and stage “x” were excluded from the chi-square test

### The relationship with MSI and TMB

**Fig. 5:**
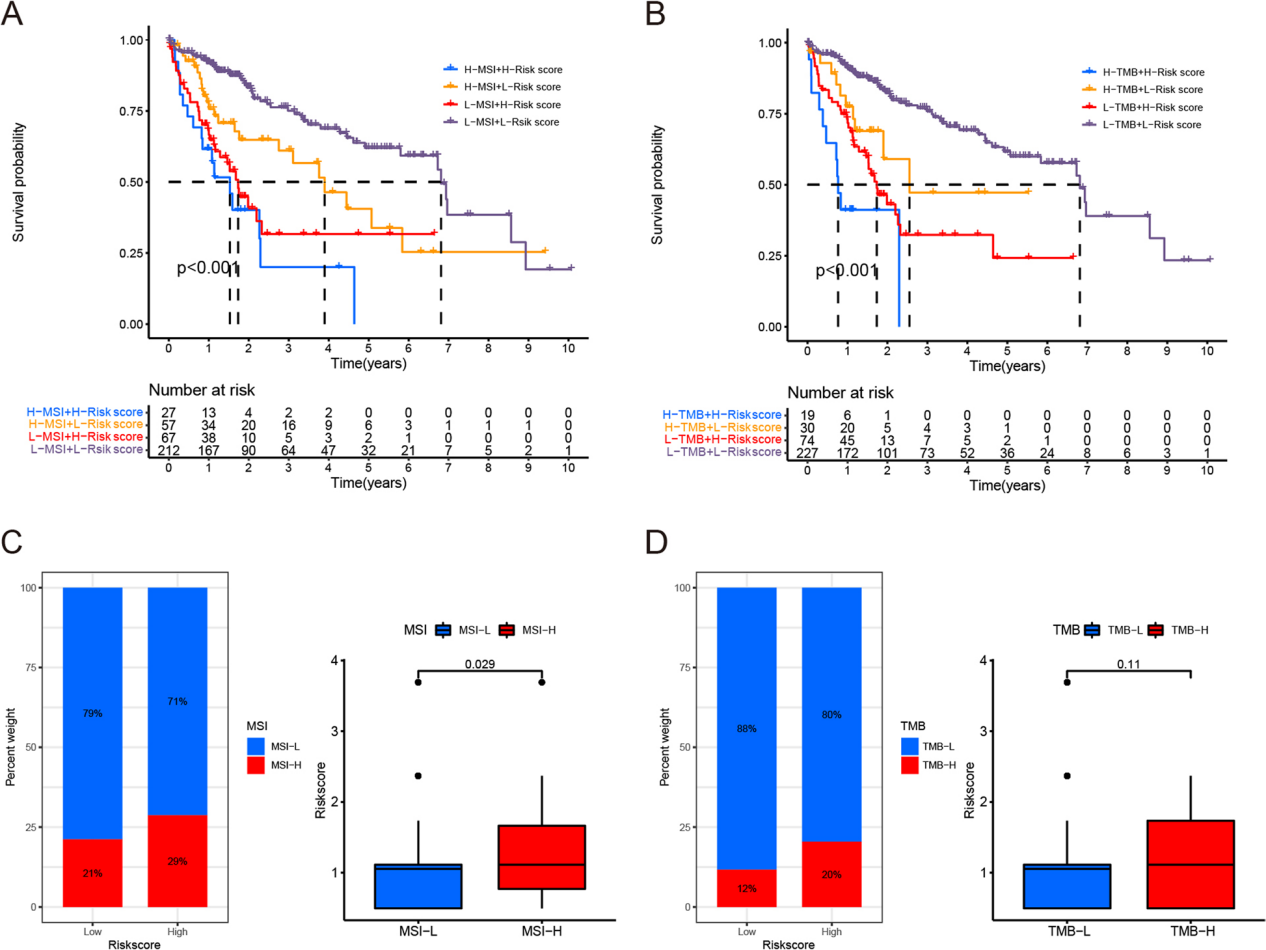
Relationship with MSI and TMB. **A**, **B** Kaplan-Meier survival analysis using the risk score combined with MSI or TMB. **C**, **D** Correlation analysis between the risk score and MSI or MTB

Combining MSI and TMB increased the predictive accuracy of the risk score even more than using either of them individually. In this study, associated with survival outcomes, we found that when the optimal cutoff of MSI (0.3295) and TMB (2.9474) were applied to divide the patients into high and low groups of MSI and TMB respectively, the difference in OS among the subgroups was the greatest (Fig. 5A, B). The hazard of the high MSI + high risk score group had the worst survival compared with the other groups (Fig. 5A). Patients with MSI-H were accounting for 29% of the high-risk group, which was higher than the 21% found for the low-risk group (Fig. 5C). Differential analysis between the MSI-L group and MSI-H group presented that the risk score was positively related to MSI (Fig. 5D). Although combined with TMB, KM survival analysis showed a significant difference among subgroups (Fig. 5B), and the high-risk score group also had a higher percentage of TMB-H than the low-risk score group (Fig. 5E), the correlation analysis between TMB and risk score did not reach a significant difference (Fig. 5F).

### Assessment of immune infiltration, sensitivity of ICIs, and targeted therapy

**Fig. 6:**
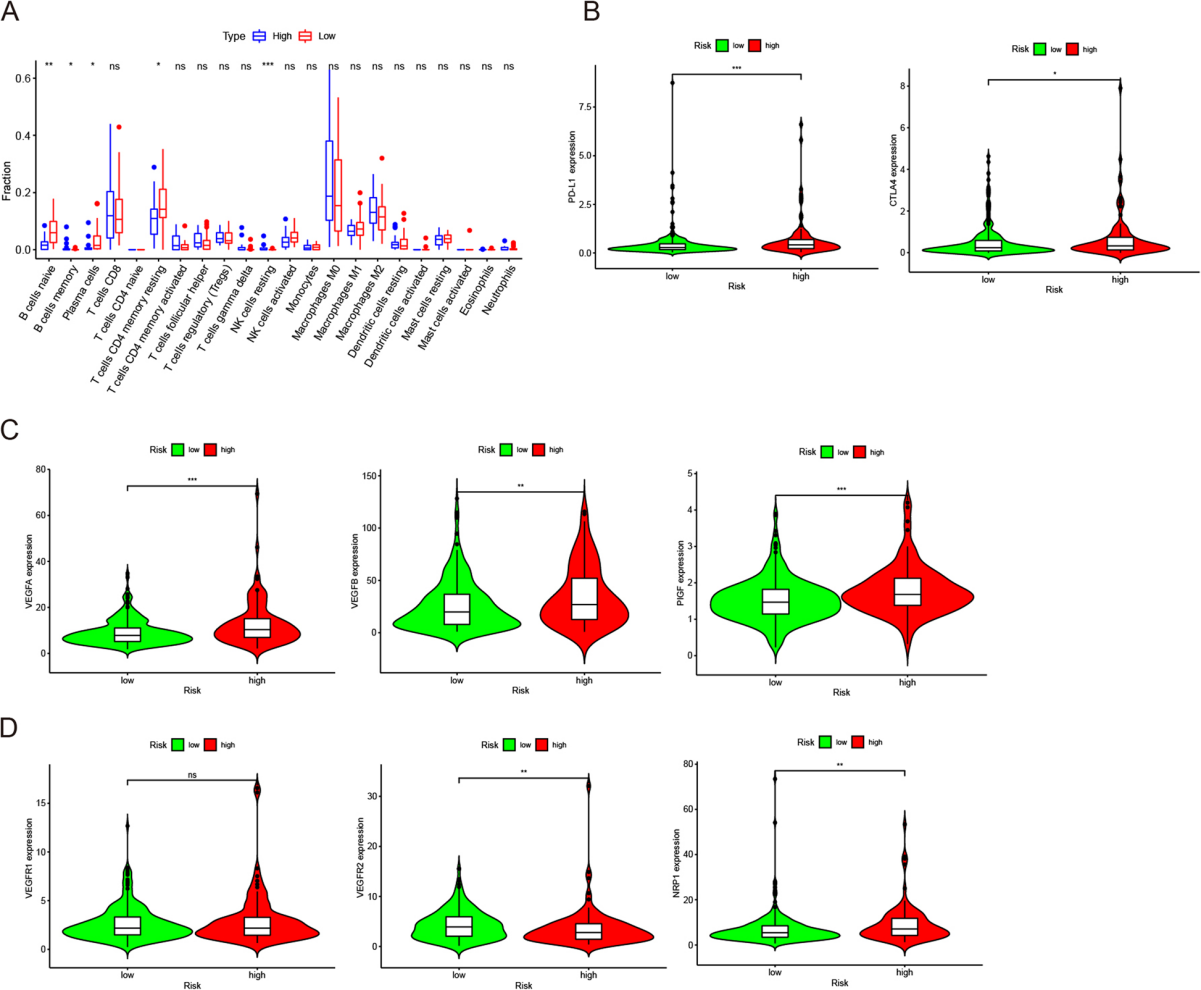
Assessment of immune infiltration, sensitivity of ICIs and target therapy. **A** Abundance of 22 different immune cells inferred by CIBERSORT for different risk groups. **B** Different expression of immune checkpoints (PD-L1 and CTLA-4) for different risk groups. **C**, **D** Different expression of VEGFA, VEGFB, PlGF, VEGFR1, VEGFR2, and NRP1 within low and high-risk groups. *p < 0.05; **p < 0.01; ***p < 0.001; ns: no significant

The immune infiltration analysis showed that 5 immune cells’ infiltration levels had a significant difference between the high and low-risk groups. The infiltration proportion of plasma cells, naive and memory B cells, and resting memory CD4^+^ T cells presented clearly higher in the low-risk group, while resting NK cells in the low-risk group had relatively high infiltration levels (Fig. 6A). This result presented that the tumor immune microenvironment is strongly associated with the risk score.

The immune checkpoints including PD-L1 and CTLA4 in the high-risk group had a clearly higher expression than that in the low-risk group (Fig. 6B). Analyses of the ligands and receptors associated with angiogenesis, VEGFA, VEGFB, and placental growth factor (PlGF) showed higher expression in the high-risk group (Fig. 6C). Although the main receptor VEGFR2 expression presented lower in the high-risk group, the expression of the regulator PlGF and its receptor neuropilin 1 (NRP-1) showed higher in the high-risk group. However, VEGFR1 showed no significant difference between both risk groups (Fig. 6D). This indicates that the risk model could help to identify patients who may have a positive response to ICIs and anti-angiogenesis target therapy.

## Discussion

HCC comprises 75–85% of liver cancers and has a poor prognosis [1]. Some studies have shown multiple genes present good predictive potential in assessing HCC prognosis [15–18]. Recently, the importance of ICI therapy in HCC patients has been proven. A combination of atezolizumab and bevacizumab has been shown to be significantly better than sorafenib for the treatment of advanced HCC [7]. However, the ORR remains unsatisfactory. Our study constructed a risk assessment model using 3 IRG pairs to explore the relationship with immune characteristics with a strong predictive ability. This might help to identify potential patients who are sensitive to this combination of immune and targeted therapy.

Since the use of gene pairs only compares the expression levels of two genes in the same sequencing batch, and not their specific expression values, this approach is different from traditional prediction models. The advantage is that the model can be verified between different batches of sequencing data without having to consider the error caused by batch correction. After constructing the model, we first tested the model’s ability to predict early survival. The results showed that the 2-year predictive value was the highest (AUC = 0.734), and that it was significantly higher than that of clinical characteristics such as tumor stage and pathological grade. Therefore, we calculated the optimal cutoff value for 2-year survival prediction. After dividing all patients into high and low-risk groups with the optimal cutoff value, the evaluation results of clinical characteristics presented that the patients in the high-risk group had worse OS rate, larger tumor size, and higher tumor stage. To evaluate the practicability of this prediction model, we selected the ICGC and GSE14520 cohort for external validations because they had large sample size and complete corresponding clinical data. As we expected, similar prediction effects were obtained for both ICGC and GSE14520, which showed that our model was reliable.

When screening the mRNAs for constructing the model, we extracted immune-related mRNAs from the immune database. These mRNAs are involved in encoding cytokines and their receptors, immune checkpoints, and other protein molecules involved in cellular immunity. The different expression of these immune-related proteins in HCC patients will change the level of immune cell infiltration in the tumor microenvironment, leading to differences in the effectiveness of immunotherapy between different individuals. The six immune-related mRNAs involved in the model construction have been reported to participate in the immune regulation and progress of malignant tumors. Collectin subfamily member 10 (COLEC10) encodes for collectin liver 1 (CL-L1) [19]. Recent studies reported that the low expression level of COLEC10 may predict poor OS in patients with HCC [20], and knock-down expression level of COLEC10 can promote liver tumor cells’ proliferation, migration and invasion in vitro [21]. COLEC10 is a protective factor in the model, and patients with higher expression levels of COLEC10 than MMP12 can have a better prognosis and first-line treatment options. Human matrix metallopeptidase 12 (MMP12) was first identified in human alveolar macrophages [22]. A high expression level of MMP12 in HCC can promote tumor FOXP3+ regulatory T cell infiltration and contribute to a poor prognosis [23]. Lymphatic spread is an important clinical determinant for the prognosis of HCC [24]. Tumors with high VEGFD expression showed increased microvessel density and an abundance of lymphatic vessels around and within the tumor [24]. Based on our analysis, a high expression of VEGFD is associated with a poor prognosis for HCC patients, which indicates that targeting VEGFD may be an alternative therapy for HCC. Progesterone-associated endometrial protein (PAEP), known as glycodelin, is a secreted immunosuppressive glycoprotein. One study showed that it can be a biomarker with an immune-modulatory function because of its high association with OS, recurrence and metastasis rate in non-small cell lung cancer [25]. For chondroitin sulfate proteoglycan 5 (CSPG5), one study mentions it could be served as a prognostic factor for breast cancer based on immunohistochemical analysis [26]. Programmed cell death 1 (PD-1) has been proven to be a target in HCC in clinical trials. Nivolumab exhibits a high affinity and specific targeting to an epitope of PD-1 [27]. The risk assessment model in our study includes PD-1, which allows the model to more accurately identify patients who are sensitive to immunotherapy and evaluate patients’ immune cell infiltration.

MSI and TMB were treated as biomarkers to assess the efficacy of immunotherapy in human malignancies [28]. Patients with MSI-H or TMB-H were linked to poorer OS, but they had higher ORR than those with MSI-L or TMB-L with ICI therapy [29]. However, the subset of patients with high MSI or TMB has not been well characterized in HCC patients [30]. In this study, we associated with survival outcomes, found the optimal cutoff of MSI and TMB, and achieved the greatest difference in the prognosis of subgroups. More interestingly, our risk score was positively associated with MSI, and negatively with TMB. This may indicate that MSI has a greater predictive power than TMB in HCC based on our model.

The expression of immune checkpoints, including PD-1, PD-L1, and CTLA-4, has been associated with tumor aggressiveness and poor prognosis [31, 32]. A high expression of PD-L1 has been correlated with the rate of response to PD-L1/PD-1 targeting therapies in most clinical trials [33], which indicated that PD-L1 can help to identify the type of patients that will benefit from ICI therapy. The risk score of our model is strongly associated with the expression of PD-L1 and CTLA-4. It seems that the high-risk group may have a better ORR compared to the low-risk group.

VEGFA plays a dominant role in regulating angiogenesis and disease, and high expression of VEGF is observed in the majority of human tumors and positively correlated with aggressiveness, metastasis, recurrence, and prognosis [34]. It can bind to both VEGFR1 and VEGFR2, while VEGFR2 is the main signaling receptor for VEGFA [35]. Moreover, heparin-binding VEGFA and PlGF can bind to NRP-1 to increase their binding affinity to VEGFR2 [36]. In this study, we found the high-risk group presented a higher expression of VEGFA. Although the VEGFR2 expression was lower in the high-risk group, the regulators PlGF and NRP-1 were expressed higher in the high-risk group which can compensate for the binding affinity to VEGFR2. This indicates that our risk model has a high association with VEGFA and it may help to assess the sensitivity of anti-angiogenesis target therapy.

Compared with other published models which only accessed the HCC prognosis [37–41], our risk score model was focused on both the prognosis and the sensitivity to the latest first-line therapy of HCC patients. And it showed a significant difference in both the prognosis and expressions of PD-L1 and VEGFA between high and low risk score groups. It only based on 3 IRG Pairs and attained a similar AUC value as the other prognostic model with 10 immune-related genes [42] and showed good quality of applicability and stability in both ICGC and GSE14520. Furthermore, it is worth pointing out that the predicting power of our model is better than that of other reported models using 6 genes [43] or 11 immune-related genes [44] (Supplementary Table 1).

This study has several limitations. First, the AUC of OS was not higher than that of other published models [37–41]. When screening the immune gene pairs to construct the model that satisfies both prognosis and significant different expressions of PD-L1 and VEGFA between high and low risk score groups, only these 3 immune-related gene pairs were selected. Generally speaking, a model with more genes and gene pairs will attain a higher AUC of OS. Second, our model did not include some earlier reported HCC prognosis genes [15–18]. Our prediction model only included 3 immune-related gene pairs with 6 genes to assess both the prognosis and treatment sensitivity to immune and targeted therapy of HCC patients, and it was hard to include all genes reported. Actually, it contained 4 genes which were associated with the prognosis of HCC [20, 23, 24, 27] and 2 genes which have been proved to be related to the prognosis of other tumors [25, 26]. Third, we only used public datasets to construct the model and external validation, the practicality should be identified and the detailed relationship should be confirmed by further experiments.

## Conclusions

Here, we constructed a risk assessment model using 3 immune-related gene pairs that is helpful for assessing the survival, immune characteristics, and therapeutic sensitivity of HCC patients.

## Supplementary Information

**Additional file 1: Supplementary Table 1.** Comparison of model signature and AUC among our risk model and other models of prognosis.

## Data Availability

The data used in the paper can be downloaded from the public databases (TCGA, ICGC, and GSE14520). If you need more details of analyzing data, please contact the corresponding author.
