# Supplementary material for: Risk predictive model based on three immune-related gene pairs to assess prognosis and therapeutic sensitivity for hepatocellular carcinoma
Source: World J Surg Oncol. 2022 Aug 5;20:252. doi: 10.1186/s12957-022-02681-4 (PMC9354375; doi:10.1186/s12957-022-02681-4)
Supplement: Supplementary file 1 — Additional file 1: Supplementary Table 1. Comparison of model signature and AUC among our risk model and other models of prognosis. [file 12957_2022_2681_MOESM1_ESM.docx]

Supplementary Table 1: Comparison of model signature and AUC among our risk model and other models of prognosis.

| Author | Year | Gene Signature | Function | AUC for OS | Average AUC for OS |
| --- | --- | --- | --- | --- | --- |
| Li WL, et al[43] | 2019 | 6 genes signature related to the prognosis | To access the HCC prognosis | 0.681 (1-year), 0.700 (3-year), 0.684 (5-year) | 0.688 |
| Dai YF,et al[44] | 2021 | 11 immune-related genes related to the prognosis | To access the HCC prognosis and the efficacy of immunotherapy | 0.789 (1-year), 0.693 (3-year), 0.663 (5-year) | 0.715 |
| Zhao KF, et al[42] | 2020 | 10 immune-related genes signature related to the prognosis | To access the HCC prognosis | 0.739 (1-year), 0.731 (2-year), 0.695 (3-year) | 0.720 |
| Our risk model |  | 3 immune-related gene pairs related to the prognosis and PD-L1 expressions | To access the HCC prognosis and the sensitivity of HCC first-line therapy | 0.716 (1-year), 0.734 (2-year), 0.711 (3-year) | 0.720 |
| Liu RJ, et al[37] | 2021 | 5 apoptosis-related genes | To access the HCC prognosis | 0.741 | 0.741 |
| Yang HB, et al[38] | 2021 | 5 genes related to ARID1A mutations | To access the HCC prognosis | 0.838 (1-year), 0.741 (3-year), 0.692 (5-year) | 0.757 |
| Huo JY, et al[39] | 2020 | 15 immune-related gene pairs associated with the prognosis and Tumor Mutation Burden | To access the HCC prognosis | 0.830 (1-year), 0.800 (3-year), 0.764 (5-year) | 0.798 |
| Chen WB, et al[40] | 2020 | 9 immune-related gene pairs related to the prognosis | To access the HCC prognosis | 0.754 (1-year), 0.847 (3-year), 0.834 (5-year) | 0.812 |
| Sun XY, et al[41] | 2020 | 33 immune-related gene pairs related to the prognosis | To access the HCC prognosis | 0.912 (1-year), 0.918 (3-year), 0.814 (5-year) | 0.881 |
